# Bisphenol A promotes stress granule assembly and modulates the integrated stress response

**DOI:** 10.1242/bio.057539

**Published:** 2021-01-11

**Authors:** Marta M. Fay, Daniella Columbo, Cecelia Cotter, Chandler Friend, Shawna Henry, Megan Hoppe, Paulina Karabelas, Corbyn Lamy, Miranda Lawell, Sarah Monteith, Christina Noyes, Paige Salerno, Jingyi Wu, Hedan Mindy Zhang, Paul J. Anderson, Nancy Kedersha, Pavel Ivanov, Natalie G. Farny

**Affiliations:** 1 Division of Rheumatology, Immunology, and Allergy, Brigham and Women's Hospital, Boston, 02115 USA; 2 Department of Medicine, Harvard Medical School, Boston, MA, 02115 USA; 3 Department of Biology and Biotechnology, Worcester Polytechnic Institute, Worcester, MA, 01609 USA; 4 Broad Institute of Harvard and MIT, Cambridge, MA, 02142 USA

**Keywords:** Bisphenol-A, Integrated stress response, Stress granules, Translational control

## Abstract

Bisphenol-A (BPA) is a ubiquitous precursor of polycarbonate plastics that is found in the blood and serum of >92% of Americans. While BPA has been well documented to act as a weak estrogen receptor (ER) agonist, its effects on cellular stress are unclear. Here, we demonstrate that high-dose BPA causes stress granules (SGs) in human cells. A common estrogen derivative, β-estradiol, does not trigger SGs, indicating the mechanism of SG induction is not via the ER pathway. We also tested other structurally related environmental contaminants including the common BPA substitutes BPS and BPF, the industrial chemical 4-nonylphenol (4-NP) and structurally related compounds 4-EP and 4-VP, as well as the pesticide 2,4-dichlorophenoxyacetic acid (2,4-D). The variable results from these related compounds suggest that structural homology is not a reliable predictor of the capacity of a compound to cause SGs. Also, we demonstrate that BPA acts primarily through the PERK pathway to generate canonical SGs. Finally, we show that chronic exposure to a low physiologically relevant dose of BPA suppresses SG assembly upon subsequent acute stress. Interestingly, this SG inhibition does not affect phosphorylation of eIF2α or translation inhibition, thus uncoupling the physical assembly of SGs from translational control. Our work identifies additional effects of BPA beyond endocrine disruption that may have consequences for human health.

## Abstract (precis)

**Summary:** Physiologically-relevant doses of the plasticizing agent BPA inhibit stress granule formation in response to a secondary acute stress, indicating BPA may affect the way human cells cope with cellular stress.

## INTRODUCTION

Bisphenol-A (BPA) is one of the highest volume chemicals produced globally, with an estimated 7.7 million metric tons produced in 2015 at an estimated value of US$15.6 billion (Research and Markets, 2016). BPA is used in the production of polycarbonate plastics and epoxy resins that are found in myriad consumer products including food packaging, receipts, automobiles, electronics, and medical devices. BPA enters the human body through ingestion or inhalation of plastic dust and particulates. A National Health and Nutrition Examination Survey (NHANES) study conducted by the Centers for Disease Control and Prevention (CDC) in 2003–2004 found that 92.6% of people tested had detectable levels of BPA in their urine (Calafat et al., 2008).

Many lines of evidence demonstrate the activity of BPA as an endocrine disrupting compound (Rochester, 2013; Ben-Jonathan, 2019). While the physiological relevance of this endocrine disruption is debated, many studies have linked BPA to infertility, obesity, diabetes, and cancer (Seachrist et al., 2016; Provvisiero et al., 2016; Qiu et al., 2016; FDA, 2018; Apau et al., 2018). The US Food and Drug Administration (FDA) currently maintains that BPA is safe at the levels detected in the general population (FDA, 2018). Still, public concern has led to pressure on manufacturers to remove BPA from consumer products. Most often, BPA is replaced with highly similar chemical analogs, such as BPS, BPF, or BPAF. Therefore, human exposure to the broader class of bisphenols is on the rise, and even less is known about the health effects of these BPA alternatives (Rosenmai et al., 2014).

An important consequence of BPA exposure that has been demonstrated in multiple contexts is oxidative stress, and the generation of reactive oxygen species (ROS) (Gassman, 2017). It is hypothesized that the metabolic breakdown of BPA may result in a greater cellular load of ROS than available antioxidants can manage, leading to oxidative damage (Gassman, 2017), and that these effects may be independent of the endocrine disrupting properties of BPA. For example, increased proliferation, ROS, and DNA damage were observed in cells lacking ER-α (Pfeifer et al., 2015). The exact mechanisms of BPA-related oxidative stress remain to be elucidated.

Stress granules (SGs) are cytoplasmic aggregates of proteins and mRNAs that form in response to stress-induced inhibition of mRNA translation (Anderson and Kedersha, 2009), and are biomarkers of the integrated stress response (Buchan et al., 2008; Farny et al., 2009; Kedersha et al., 1999). SGs are conserved among eukaryotic organisms from yeast to humans and are triggered by a wide variety of extracellular stresses including energy starvation, ion imbalance, oxidative stress, heat shock, and UV irradiation. The composition and dynamics of SGs vary based on the stressor (Aulas et al., 2017). SGs are generally thought to be protective, and conserve cellular resources by silencing mRNA translation during stressful circumstances. In some instances, SGs delay the onset of apoptosis in response to acute cellular stress, and the disruption of SGs can make cells more vulnerable to death (Arimoto et al., 2008). Indeed, the cellular consequences of endocrine disruption, such as oxidative stress, are also triggers for SG assembly, though direct links between endocrine disrupting compounds and SGs have yet to be examined.

SGs are a downstream consequence of a broader cellular stress response known as the integrated stress response. The integrated stress response is a mechanism by which a variety of environmental stresses (e.g. oxidative stress, UV irradiation, toxins), or biotic stresses (e.g. fever, viral infection) can signal the translation machinery to decrease protein synthesis, thereby conserving energy and permitting the cell to redirect resources to survival and the activation of various downstream stress response pathways (Pakos-Zebrucka et al., 2016). In mammalian cells, there are four kinases that can be activated during the integrated stress response: heme–regulated eIF2α kinase (HRI), double-stranded RNA-dependent protein kinase (PKR), general control non-depressible kinase 2 (GCN2), and PKR-like endoplasmic reticulum kinase (PERK). Some stresses specifically activate one of the four kinases. For example, sodium arsenite specifically activates HRI (McEwen et al., 2005), and viral infection activates PKR (Garcia et al., 2006). These kinases once activated all target the same mechanism, the phosphorylation of serine 51 of the alpha-subunit of eIF2, which blocks the formation of initiation ternary complex (eIF2-GTP-tRNA^MET^) and thereby results in translational arrest. Often, the phosphorylation of eIF2α-Ser51 triggers the assembly of SGs (Kedersha et al., 2002), though SGs can also form through eIF2-independent mechanisms (Aulas et al., 2017; Farny et al., 2009). Whether BPA activates any of these kinase pathways was previously unknown.

Here, we investigated the link between the endocrine-disrupting compound BPA and SGs. We determined that high doses of BPA cause canonical, phospho-eIF2α-dependent SG assembly via activation of PERK, one of the four eIF2α kinases that mediate the integrated stress response. The common estrogen supplement β-estradiol does not trigger SG assembly, indicating that it is unlikely that BPA-induced SG assembly is related to its reported endocrine disrupting activity. While BPA induces robust SG assembly, closely related BPA substitutes BPF and BPS cause lower or no SG assembly, respectively. Similarly, we find that the industrial chemical 4-nonylphenol (4-NP), but not highly related structural compounds 4-EP and 4-VP, trigger SG assembly, indicating that structural homology is not a good predictor for promoting SG assembly. Finally, we show that long term, low-dose BPA exposure within the physiologically relevant range can alter SG dynamics by suppressing SG assembly upon later acute stress exposures. Importantly, chronic BPA treatment does not affect phosphorylation of eIF2α or translation inhibition, therefore uncoupling the physical assembly of SGs from eIF2α-mediated translational control. Our results suggest that real-world chronic BPA exposure may compromise the ability of human cells to cope with environmental stress.

## RESULTS

### High levels of BPA induce SGs but not processing bodies (PBs)

**Fig. 1.**
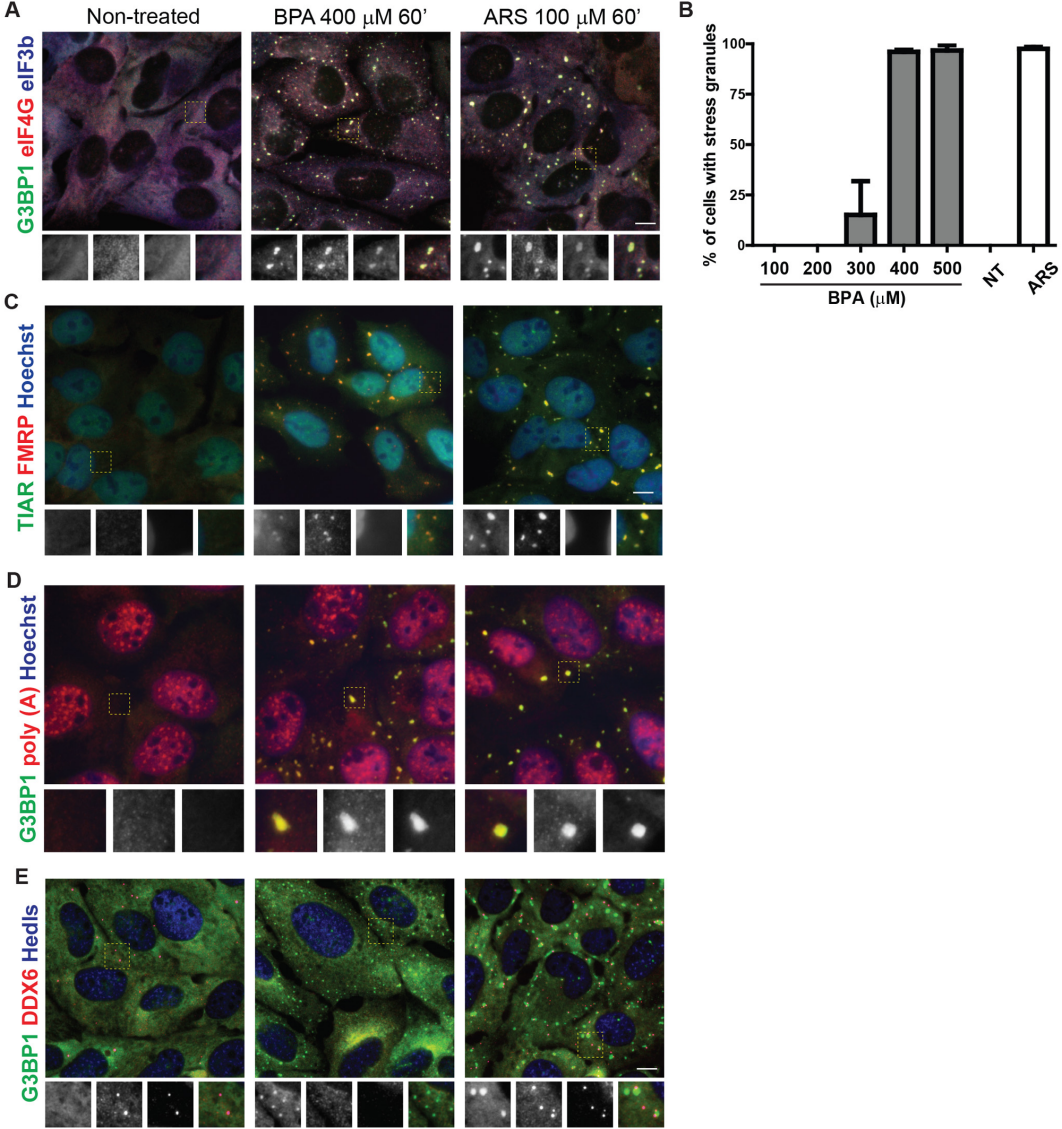
**BPA promotes SG formation.** (A) Immunofluorescence of U2OS cells untreated (left) or treated with BPA (400 µM) (center) or arsenite (ARS, 100 µM) for 1 h (right) detecting G3BP1 (green), eIF4G (red), eIF3b (blue). Boxed area is shown zoomed at 1.8X of original and individual channels are shown as black and white in order of green, red, blue then merged as a RGB image. (B) U2OS cells were treated with the indicated range of BPA concentrations or 100 µM ARS for 1 h, or left untreated (NT) then assessed for SG formation by immunofluorescence. Quantification of SGs was determined by the total number of cells containing two or more G3BP1 positive foci over the total number of cells. (C) Same as A, but detecting TIAR (green), FMRP (red), and Hoechst (blue). (D) U2OS cells treated as indicated in B then fluorescence *in situ* hybridization to detect poly (A) RNAs (red) followed by immunofluorescence to detect G3BP1 (green) and counterstained with Hoechst (blue). (E) Same as B but detecting G3BP1 (green), DDX6 (red), and Hedls (blue). Scale bar indicates 10 microns.

BPA has been associated with several potential health risks (Apau et al., 2018; Seachrist et al., 2016; Qiu et al., 2016; Provvisiero et al., 2016). We hypothesized that BPA may trigger the integrated stress response, and used SG assembly as a biomarker. We treated cells with a range of BPA concentrations and exposure times, and used fluorescence microscopy to detect intracellular localization of the canonical SG component G3BP1. We found that G3BP1-positive SGs form in ∼15% of U2OS cells after treatment with 300 µM BPA for 60 min (Fig. 1A–B). Treatment with 400 µM BPA for 60 min consistently induced granules in ∼100% of cells (Fig. 1A–B), therefore this concentration was used throughout further analyses.

While G3BP is a widely-accepted marker for SGs, we employed other criteria to confirm that these BPA-induced G3BP foci were canonical SGs. To determine whether their composition was consistent with canonical SGs, cells were treated with BPA or sodium arsenite (ARS) as a positive control, then immunofluorescence was used to assess for colocalization of other known SG markers with G3BP1. The translation initiation factors eIF4G and eIF3b colocalize with G3BP1 at cytoplasmic BPA-induced granules (Fig. 1A). The RNA-binding proteins and classical SG markers fragile X mental retardation protein (FMRP) and T-cell intracellular antigen related protein (TIAR) colocalize in BPA-induced granules (Fig. 1C).

SGs contain polyadenylated [poly(A)] mRNA (Kedersha et al., 1999). We used fluorescence *in situ* hybridization (FISH) with an oligo dT probe to assess poly(A) RNA recruitment to BPA-induced granules. Poly(A) RNA consistently colocalized with BPA-induced G3BP1-positive foci in a similar manner to ARS-induced SGs (Fig. 1D), indicating poly(A) mRNAs are localized to BPA-induced granules. Together these data indicate that high doses of BPA induce cytoplasmic foci that contain poly(A) RNA, G3BP1, FMRP, FXR1, eIF3b, eIF4G, and TIAR, which is entirely consistent with BPA-inducing canonical SGs.

PBs are another cytoplasmic RNA granule assembled under some stress conditions (Kedersha et al., 2005). We assessed whether BPA also promotes PB formation via immunofluorescence using the PB markers Hedls and DDX6. We observed little to no focus formation of Hedls (also known as EDC4) or DDX6 in BPA treated cells, in contrast to expected foci formation in ARS treated cells (Fig. 1E), indicating that unlike ARS, BPA does not promote PB formation.

### High doses of BPA disrupt translation and promote SG condensation

**Fig. 2.**
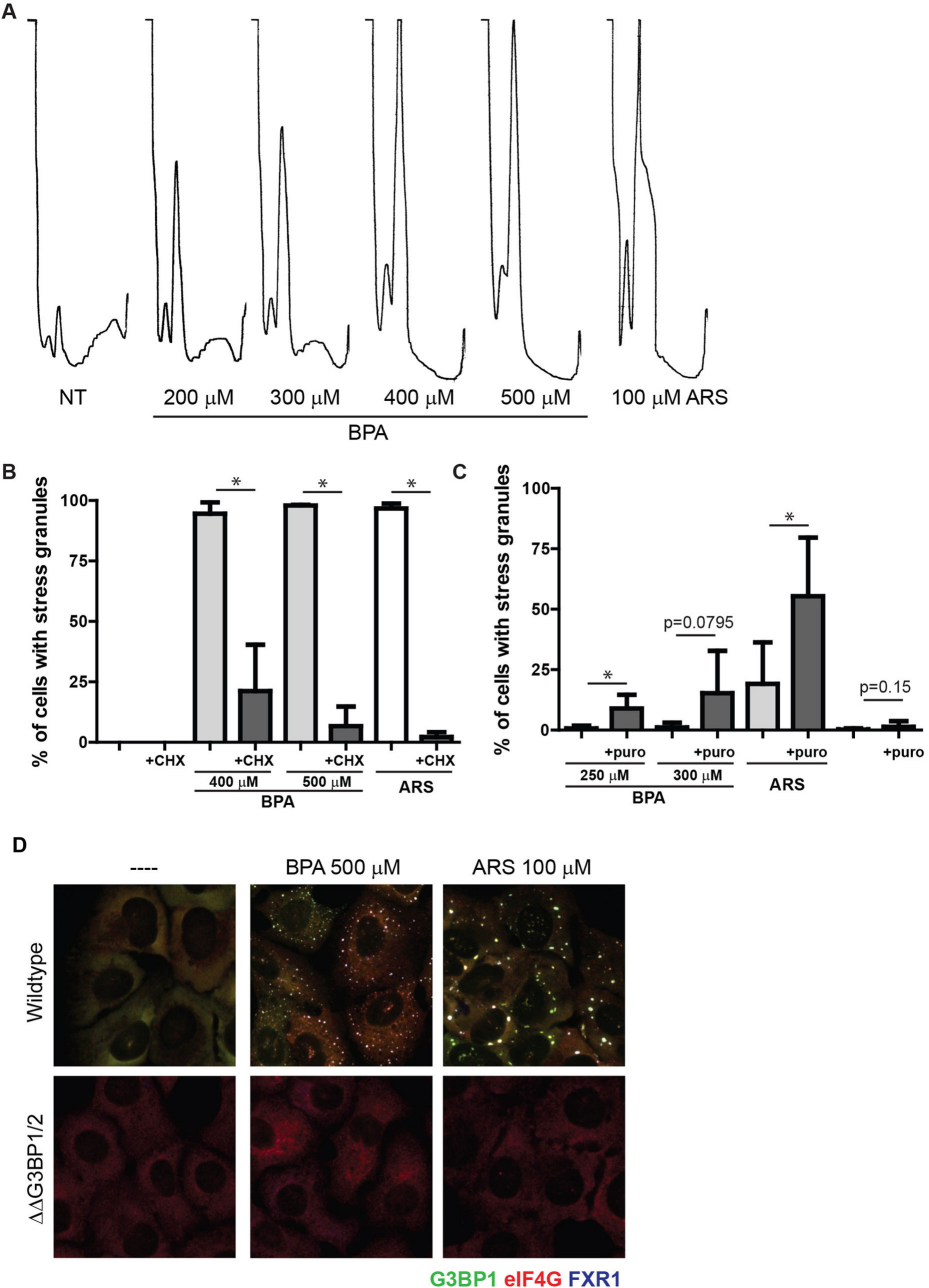
**BPA induced SGs requires polysome disassembly and G3BP1/2 mediated condensation.** (A) Polysome profiles of U2OS cells treated with indicated concentrations of BPA or ARS for 60 min. Profiles were generated using OD 254 nm. (B) U2OS cells were treated with BPA or ARS (100 µM) for 60 min. Where indicated, 50 µg/ml of cycloheximide was added for the final 30 min. Immunofluorescence was performed and used to quantify the percentage of cells with SGs. Scale bar indicates 10 microns. (C) U2OS cells were treated with indicated concentrations of BPA or ARS (50 µM) for 60 min, and co-treated with 20 µg/mL puromycin where indicated. Immunofluorescence analysis was used to quantify the percentage of cells with SGs. (D) G3BP1 (green), eIF4G (red), and FXR1 (blue) in wild-type U2OS cells (top panel), or U2OS cells lacking G3BP1/2 (ΔΔG3BP1/2; bottom panel) treated with indicated concentration of BPA or ARS for 60 min or left untreated. (B,C) Graphs represent mean with standard deviation; *n*≥3; * 0.05≥*P*.

Canonical SGs form upon translation initiation arrest and require G3BP1 and/or G3BP2 for their formation (Kedersha et al., 2016). To assess the effects of BPA on the translational status of cells, we used polysome profiling, a method of monitoring the relative translational state by density centrifugation, followed by measuring optical density at 254 nm across the eluted gradient. Treatment with SG-inducing levels of BPA caused collapse of polysome peaks, indicative of highly translated transcripts binding multiple ribosomes, and a corresponding increase in monosomes, or mRNAs that are initiated or being translated by one ribosome (Fig. 2A). This is consistent with the effect observed with SG-inducing levels of ARS (Fig. 2A).

Canonical SGs are in dynamic equilibrium with polysomes. Experimentally, this can be assessed by blocking translocation of 40S subunits using the translation elongation inhibitor cycloheximide, which stabilizes polysomes and decreases SG formation, or puromycin, which promotes premature ribosome termination and polysome disassembly, increasing dissociation into 40S subunits that are components of SGs, thereby promoting SG formation (Kedersha et al., 2000). Cycloheximide treatment blocked BPA-induced SG assembly (Fig. 2B), and conversely, puromycin treatment promoted BPA-induced SG assembly at lower concentrations of BPA (Fig. 2C). Similar results were observed with the canonical SG inducer ARS (Fig. 2B,C).

The second step in SG assembly is condensation of SG components by the proteins G3BP1/2. G3BP1/2 are required for canonical SG formation downstream of polysome disassembly, and loss of both proteins prevents SG formation (Kedersha et al., 2016). To further characterize BPA-induced granules as canonical SGs, we treated SG-incompetent G3BP1/2 knockout U2OS cells with SG promoting levels of BPA, and confirmed that these cells lack the ability to form eIF4G/FXS1 SGs, as do wild-type U2OS cells (Fig. 2D, center panels). Similar results were observed with ARS (Fig. 2D, right panels). Together these data confirm that BPA induces translation initiation arrest and requires G3BP1/2 for the condensation of canonical SGs.

### PERK phosphorylation of eIF2α is required for BPA-induced SG assembly

**Fig. 3.**
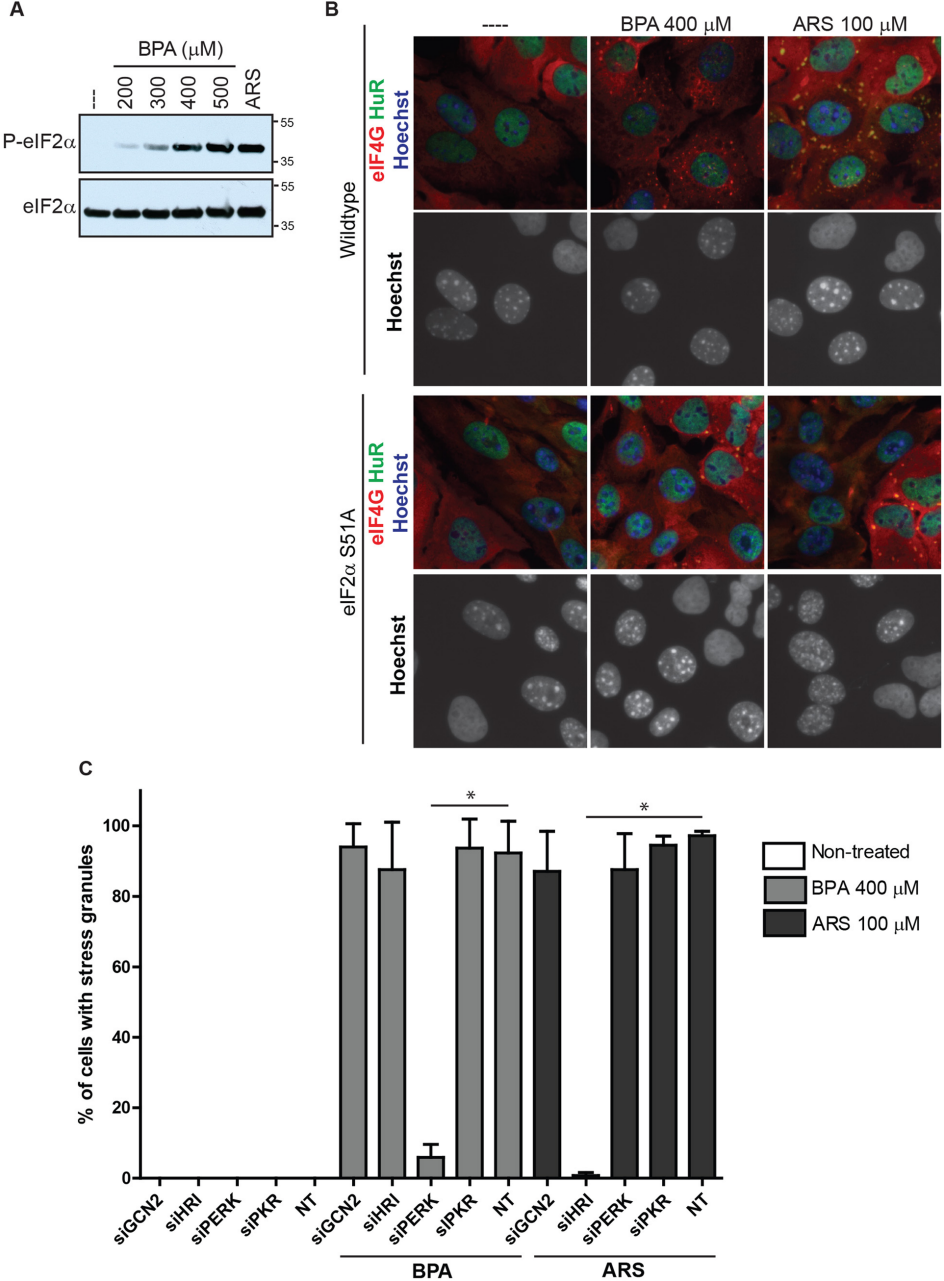
**BPA-induced SG assembly requires PERK mediated phosphorylation of eIF2α.** (A) Immunoblot detecting levels of phosphorylated eIF2α (top blot, labeled P-eIF2α) and total eIF2α (bottom blot) in U2OS cells treated as indicated with BPA, ARS (100 µM) for 1 h, or left untreated (indicated as ---). (B) Immunofluorescence detecting HuR (green), eIF4G (red), and Hoechst/DNA (blue) in wild-type MEFs co-cultured with U2OS cells (top panels), or MEFs with eIF2α-S51A point mutation co-cultured with U2OS cells (bottom panels), treated as indicated with BPA or ARS for 1 h or left untreated. MEFs exhibit punctate nuclear DNA. Scale bar indicates 10 microns. (C) Quantification of SGs in U2OS cells following siRNA-mediated knockdown of GCN2, HRI, PERK, and PKR, prior to 1 h BPA or ARS treatment, or no treatment. Graph represents mean with standard deviation; *n*≥3; * 0.05≥*P*.

Blocks in translation initiation can occur via several stages, one of which includes phosphorylation of eIF2α by stress-activated kinases. In normal translation, the eIF2α complex brings the initiator tRNA^Met^ to the AUG start codon, and phosphorylation of eIF2α prevents GTP/GDP exchange and necessary activation of the eIF2/GTP/ tRNA^Met^ complex that recruits initiator tRNA^Met^ (Sonenberg and Hinnebusch, 2009), thus blocking initiation. U2OS cells treated with a range of BPA concentrations displayed modest eIF2α phosphorylation at 300 µM, increasing to robust levels at 400 µM, comparable to those induced by 100 µM ARS (Fig. 3A). This data is consistent with the levels required to trigger SG assembly (Fig. 1A).

To assess whether eIF2α phosphorylation is required for BPA-induced SG assembly, we used mouse embryonic fibroblasts (MEFs) harboring a mutation that prevents eIF2α phosphorylation (eIF2α-S51A). Wild-type or eIF2α-S51A MEFs (distinguishable from human cells owing to speckled chromatin/Hoechst staining) were co-plated with U2OS cells, which serve as an internal control, and then treated with SG-inducing levels of BPA or ARS. SGs were observed in wild-type MEF and U2OS cells but not in eIF2α-S51A MEFs treated with BPA or ARS (Fig. 3B), indicating that eIF2α phosphorylation is required for BPA-induced SG formation.

To assess which eIF2α kinase is activated by SG-inducing levels of BPA, eIF2α kinases GCN2, HRI, PERK, or PKR were knocked down in U2OS cells with siRNA, then treated with SG-inducing levels of BPA or ARS as a control. Knockdown of GCN2, HRI, and PKR did not inhibit SG formation, but it was greatly decreased in cells treated with PERK siRNAs (Fig. 3C). Similarly, using a panel of human haploid (Hap1) cell lines with CRISPR/Cas9-mediated deletions of GCN2, HRI, PERK, or PKR (Aulas et al., 2017), we consistently found that cells lacking PERK failed to form SGs when treated with BPA (Fig. S1). Together these data indicate that BPA activates PERK to phosphorylate eIF2α and subsequently promote SGs assembly.

### BPF, 4-NP, and 2,4-D but not BPS, β-estradiol, 4-EP or 4-VP promote SGs

**Fig. 4.**
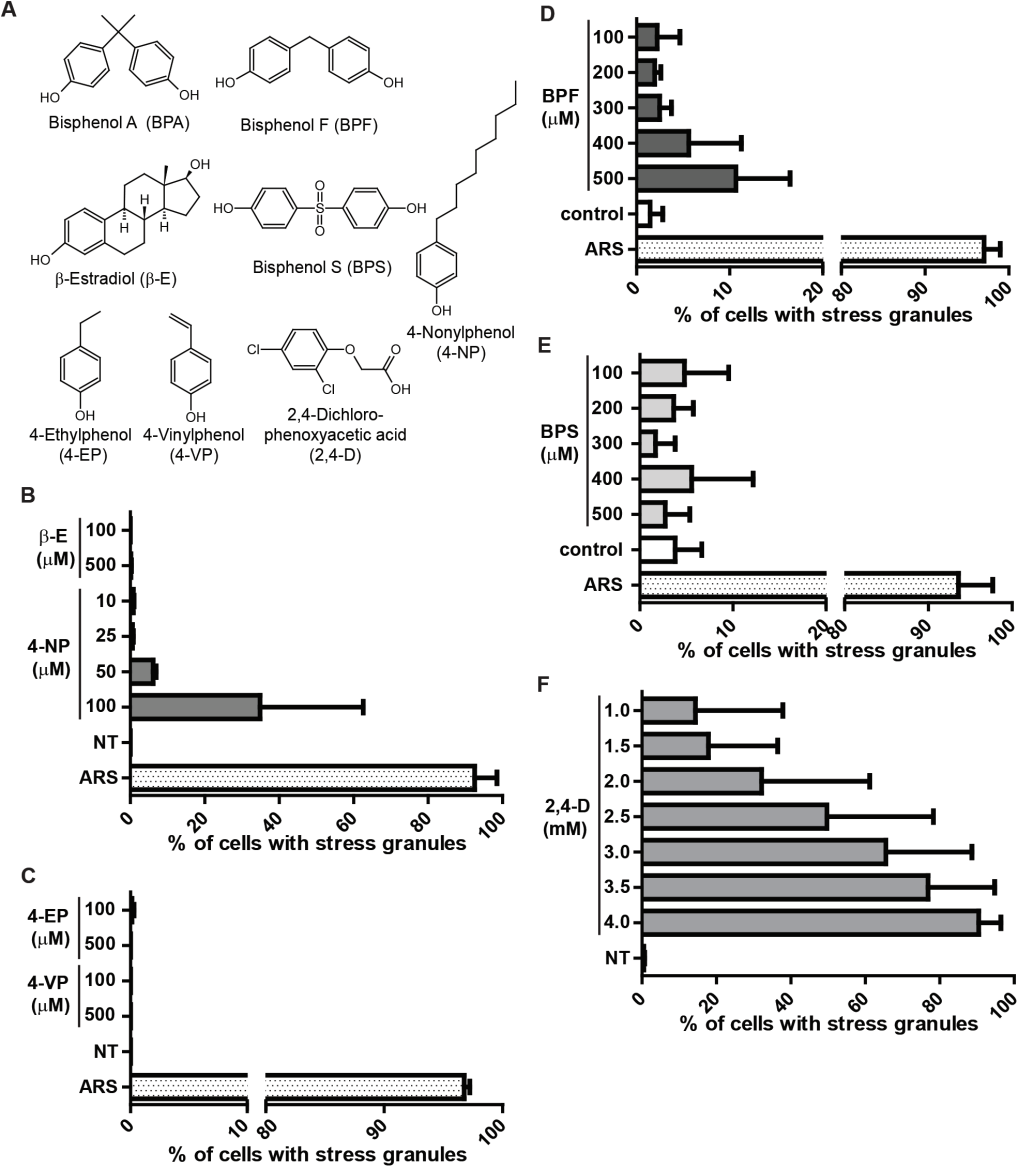
**The BPA replacement BPF, the industrial chemical 4-NP, and the pesticide 2,4-D cause SG assembly.** (A) Molecular structures of chemicals used. Structures generated using ChemSketch. (B) Quantification of SGs assessed by immunofluorescence in U2OS cells treated with indicated concentrations of β-estradiol (β-E), 4-nonylphenol (4-NP), or ARS for 1 h or left untreated (NT). (C) Same as B, but treated with 4-ethylphenol (4-EP), or 4-vinylphenol (4-VP). (D–F) Quantification of SGs assessed by direct fluorescence GFP-G3BP1 in stably expressing U2OS cells treated with indicated concentrations of BPF, BPS, 2,4-dichlorophenoxyacetic acid (2,4-D), or ARS for 1 h or mock treated (control). (B–F): Graphs represent means with standard deviation; *n*≥3; * 0.05≥*P*.

It is well documented that BPA acts as an estrogen mimic and this contributes to its adverse effects (Rochester, 2013; Ben-Jonathan, 2019). β-estradiol (β-E) is an estrogen derivative that is generated from soy and often used in as a treatment for menopause. To assess whether β-E mimics BPA in promoting SGs, U2OS cells were treated with a range of β-E concentrations and assessed for SGs by immunofluorescence. Even at high concentrations β-E does not promote SG assembly (Fig. 4B). U2OS cells are not estrogen-responsive, and do not express ER-α or ER-β receptors (Tee et al., 2004), indicating that BPA likely does not work through an estrogen pathway to promote SG assembly in U2OS cells.

Like BPA, 4-nonylphenol (4-NP) is weakly estrogenic, used industrially in detergents and food packaging, and typically detected in human samples of urine and serum (Asimakopoulos and Thomaidis, 2015; Li et al., 2013; Calafat et al., 2005). We observed G3BP1, eIF4G, eIF3b positive granule form at 50–100 µM concentrations of 4-NP (Fig. 4B). Treating with higher doses of 4-NP promoted SG formation but also caused detachment and presumably death of U2OS cells (data not shown).

Structurally, 4-NP is a phenol ring with a nine-carbon chain, and similar phenol derivatives are found naturally including 4-ethylphenol (4-EP) and 4-vinylphenol (4-VP). 4-EP and 4-VP differ from 4-NP in the length of their carbon tails (Fig. 4A). 4-EP and 4-VP are found as byproducts of bacterial metabolism and can be found at relatively high concentrations in beer and red wine (Langos and Granvogl, 2016; Teixeira et al., 2015). To test whether 4-EP or 4-VP promote SG assembly, U2OS cells were treated with a range of 4-EP and 4-VP concentrations and assessed for SG formation by immunofluorescence. Neither 4-EP nor 4-VP promoted SG formation at high concentrations (Fig. 4C), suggesting that longer carbon tails are required for SG formation.

Much public attention has been drawn to the potential adverse health effects associated with BPA, which has led to changes in BPA usage. Yet BPA is often replaced with bisphenol derivatives that remain largely untested (Rosenmai et al., 2014). We tested two commonly used BPA substitutes, bisphenol S (BPS) and bisphenol F (BPF). U2OS cells stably expressing GFP-G3BP1 were treated with a range of BPS or BPF concentrations then assessed by direct fluorescence for GFP-G3BP1 foci. Cells treated with BPF induced GFP-G3BP1 foci and quantification indicated considerably fewer cells assembled SGs compared to BPA (Fig. 4D) while BPS did not induce GFP-G3BP1 positive foci at the concentrations tested (Fig. 4E). Phosphorylation of eIF2α was observed in BPA and BPF treated cells in proportion to their degree of SG assembly, and was not observed in response to BPS treatment (Fig. S2).

Lastly, we tested whether a commonly used pesticide, 2,4-dichlorophenoxyacetic acid (2,4-D) causes SG assembly. U2OS cells that stably express GFP-G3BP1 were treated with a range of 2,4-D concentrations and found to promote SGs between 1–4 mM concentration (Fig. 4F). Together, these data indicate that like BPA, other environmentally relevant chemicals including 4-NP, BPF, and 2,4-D cause SG formation.

### Chronic low levels of BPA suppress the SG response

**Fig. 5.**
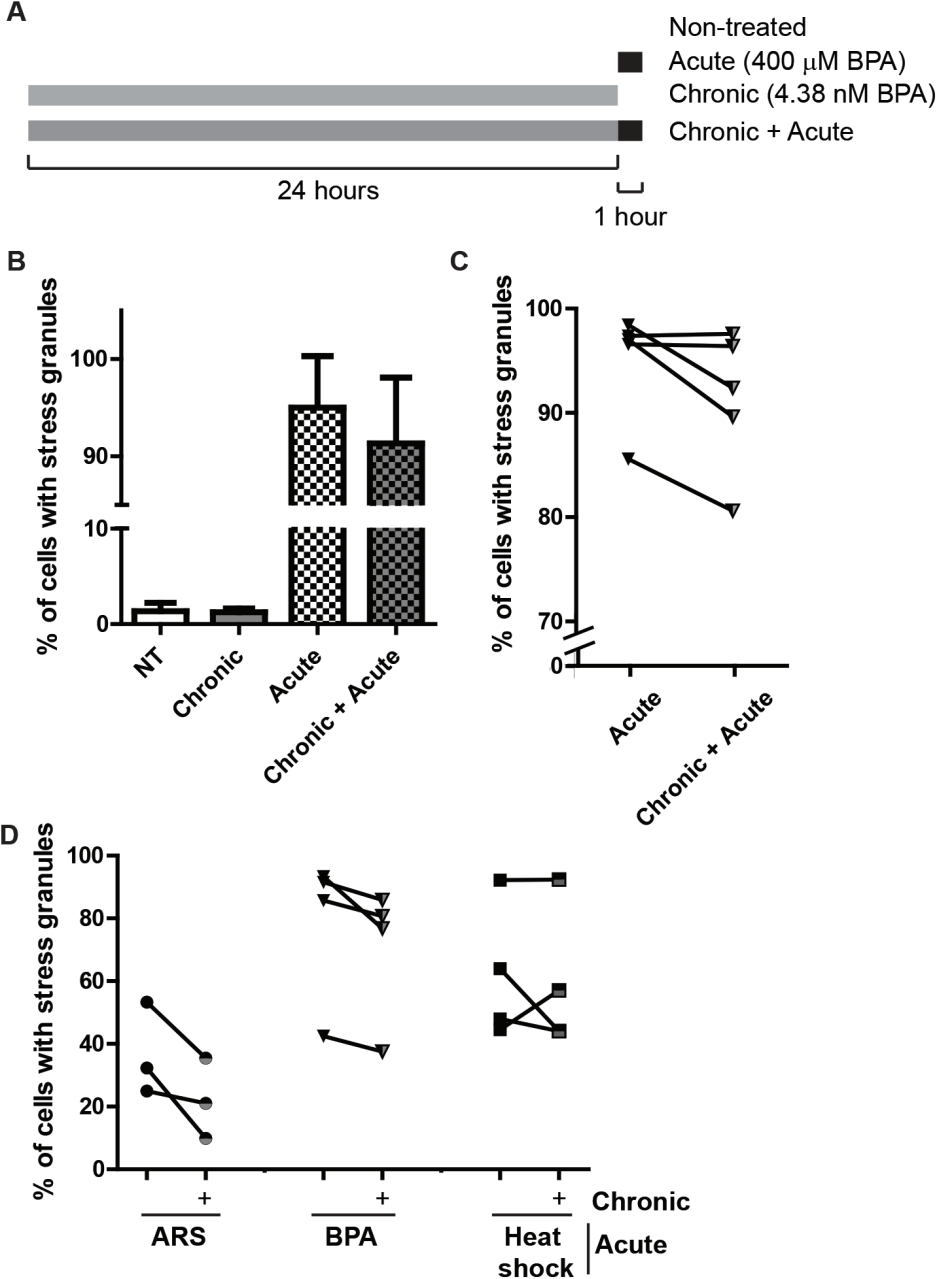
**Chronic BPA exposure using physiologically relevant doses partially inhibits SGs assembly.** (A) Schematic indicating experiment performed to generate data in B and C. (B) Quantification of SGs assesed by direct fluorescence of GFP-G3BP1 in stably expressing U2OS cells treated with BPA by acute (400 µM, 60 min), chronic (4.38 nM, 24 h) or both chronic and acute treatment. (C) Same experiment (and data) as B showing how experimental samples pair as indicated by the line. Error bars in B and C represent +/- one standard deviation. (D) Quantification of SGs detected by immunofluorescence in U2OS long term, chronically BPA-treated cells (5 nM, 1 month; half grey half black shapes) or cells left untreated (solid black shapes) then treated with 75 µM ARS or 350 µM BPA for 60 min or heat shocked at 42°C for 25 min. Lines between chronically BPA-treated and untreated samples indicate experimentally paired samples. *n*=3–5 replicates per condition, as indicated by paired data in C and D.

We have shown that high levels of BPA promote robust assembly of canonical SGs (Figs 1–3), yet these high levels of BPA are rarely observed outside of industrial settings. Low levels of BPA are observed in most of the population (90–99% of individuals tested), ranging from 1–240 nM, with an average from 5–10 nM (Calafat et al., 2005). To assess how a chronic, low dose (referred to as chronic treatment) exposure to BPA affects the cellular stress response, U2OS cells stably expressing GFP-G3BP1 were cultured in the presence of low BPA (5 nM) or left untreated for 24 h then assessed for SG formation with SG inducing levels of BPA. When challenged with a SG-inducing level of BPA, cells that were treated chronically with BPA displayed an inconsistently decreased ability to form SGs compared with cells that were not treated with chronic BPA, which fell short of statistical significance (Fig. 5B,C). It should be noted that chronic BPA treatment does not induce the formation of G3BP1 positive foci (Fig. 5B).We then extended the duration of low BPA treatment to one month to assess long-term chronic exposure. As seen with the 24 h treatment, long-term chronic treatment did not promote SGs (data not shown). Yet, challenging chronically treated BPA cells with acute arsenite or high-dose BPA reduced SG assembly (Fig. 5D).

**Fig. 6.**
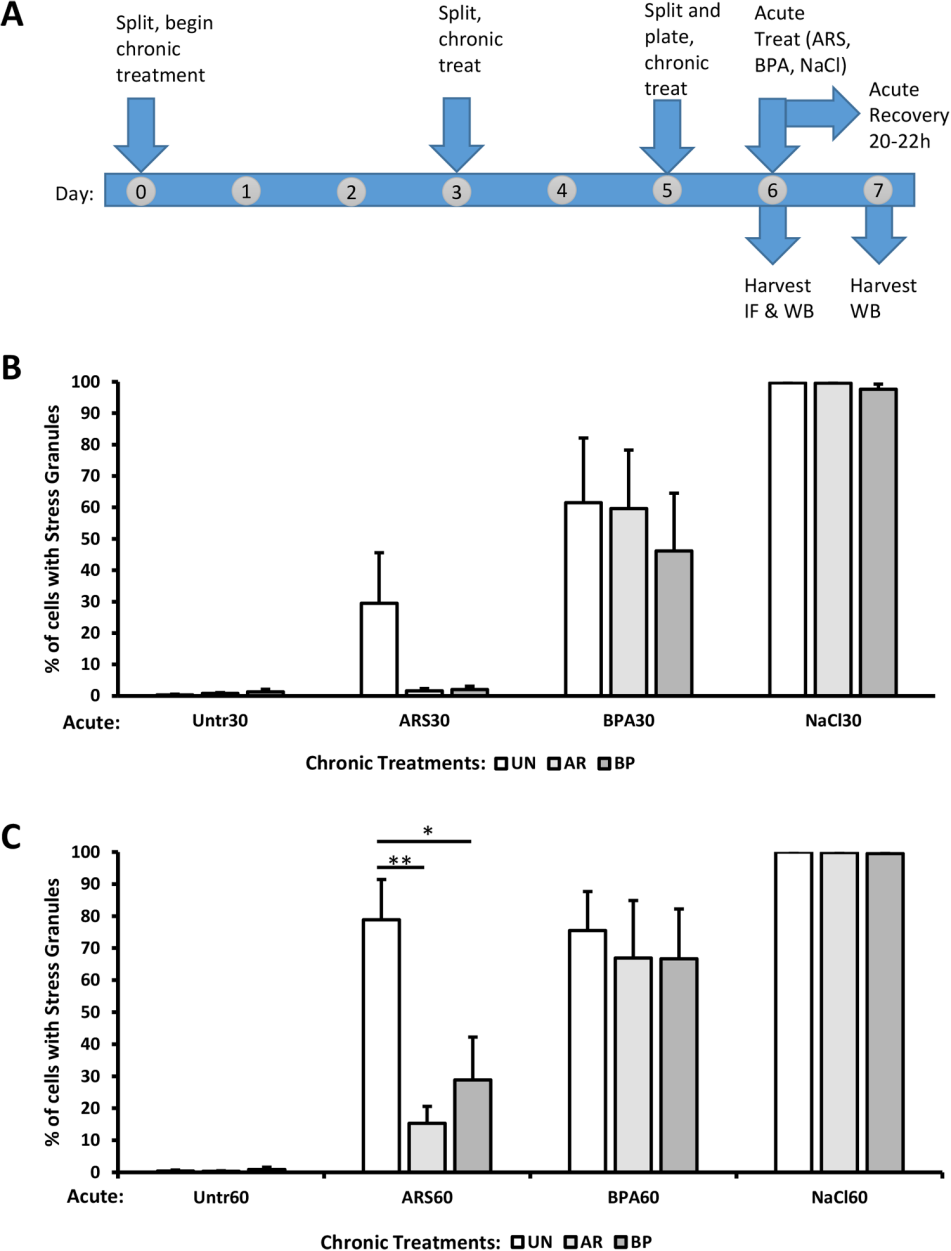
**Chronic BPA or ARS suppresses eIF2α-dependent SG formation.** (A) Schematic of the protocol for experiments in Figs 6B,C and 7. Cells were not treated (UN, white bars) or chronically treated for 6 days with 500 nM arsenite (AR, light grey bars), or 10 nM BPA (BP, dark grey bars). After chronic treatment protocol, acute stresses (100 μM arsenite, 400 μM BPA, or 0.2 M sodium chloride) were applied for 30 min (B) or 60 min (C), followed by fixation or processing for western blotting. An additional experimental replicate was treated for 60 min with acute stress and then recovered for 20-22 h before processing for western blotting or Coomassie stain (see Fig. S4). *n*=4; error bars are ±s.e.m.; *P<0.05, **P<0.01 by one-way ANOVA within acute treatment groups.

To investigate the effects of chronic exposure on BPA-mediated SG assembly further, we used a 6-day treatment protocol (Fig. 6A) to expose U2OS cells to physiologically relevant levels of BPA (10 nM) and ARS (500 nM). On day 6, we challenged these cells with several different acute stresses for 30 min (Fig. 6B) or 60 min (Fig. 6C): ARS (100 µM) which activates the eIF2α kinase HRI (McEwen et al., 2005), BPA (400 µM) which activates PERK (Fig. 3C and Fig. S1), or hyperosmotic shock (NaCl, 0.2 M) which triggers SG assembly through an eIF2α-independent mechanism (Aulas et al., 2017). A 20 hr post-stress recovery period (with chronic stressors) was included to assess post-stress translational levels.

If the SG suppression phenotype was a general feature of chronic contaminant exposures, then SGs might be suppressed by both eIF2α-dependent and -independent SG mechanisms. We observed that both chronic ARS and chronic BPA exposure dramatically suppressed SG formation in response to acute ARS treatment (Fig. 6B–C), but not significantly in response to acute BPA treatment. The SG suppression by chronic ARS and chronic BPA treatments in response to acute BPA was modest and lacked statistical significance. Notably however, SG suppression was not observed when chronically treated cells were challenged with acute osmotic (NaCl) stress. These results suggest that the SG suppression effect of both chronic ARS and chronic BPA is eIF2α-dependent, but not specific to the eIF2α kinase pathway associated with the chronic treatment. Together these data indicate that chronic treatment with low, physiologically relevant levels of BPA or ARS can affect the cellular stress response by suppressing acute ARS-induced SG assembly.

**Fig. 7.**
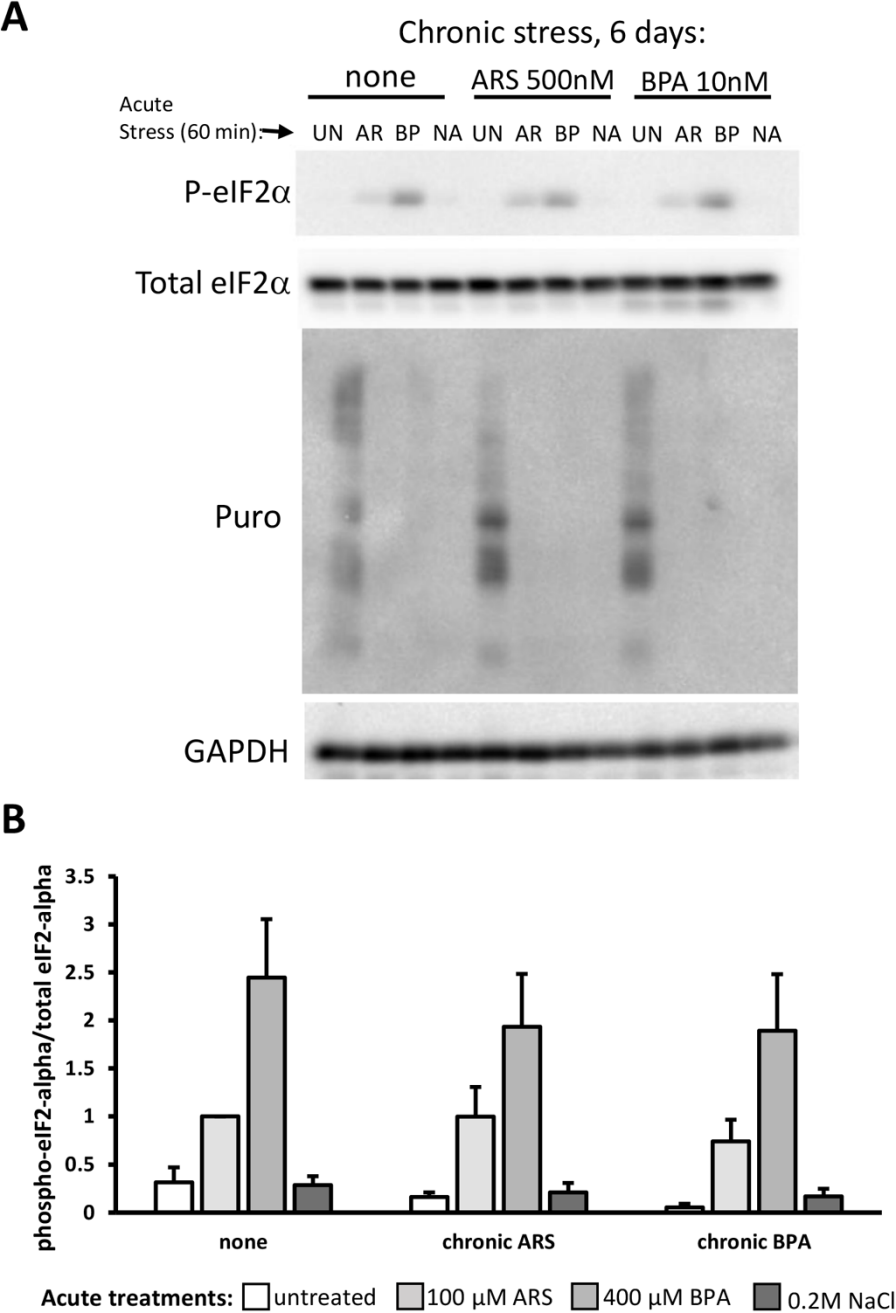
**Chronic BPA or ARS exposure does not affect eIF2α phosphorylation or translational arrest.** (A,B) Western blotting (A) of U2OS cells treated with chronic stress for 6 days (none, 500 nM arsenite, 10 nM BPA), followed by acute stress treatments with 100 μM arsenite (AR, light grey bars), 400 μM BPA (BP, medium grey bars) or 0.2 M sodium chloride (NA, dark grey bars) or untreated (UN, white bars). Puromycin (5 µg/ml) was added to the last 5 min of the 60-min acute treatment before harvesting cells for western blotting with the antibodies indicated. (B) Quantification of the phospho-eIF2α blot relative to total eIF2α. No significant difference in the pattern of responses among chronic treatments was observed by two-way ANOVA; *n*=3; error bars are ±s.e.m..

Next, we sought to determine whether eIF2α-phosphorylation or translational arrest were affected during SG suppression by chronic stress and thus account for SG suppression. U2OS cells were exposed to chronic ARS or chronic BPA treatment, followed by acute ARS, BPA, or NaCl for 60 min, and then harvested for western blot analysis. Puromycin, a translation termination factor analog that is covalently added to growing polypeptides (and thereby causes their termination), was added to the medium 5 min before harvest to assess the translational status of the cells. Surprisingly, we observed no change in expected eIF2α phosphorylation attributable to chronic treatments (Fig. 7A,B). Importantly, we observed full translational arrest in response to acute stresses regardless of the chronic treatment condition (Fig. 7A). Therefore, the suppression of SG assembly caused by chronic ARS and chronic BPA has uncoupled SG assembly from eIF2α-mediated translational control.

To examine the more widespread effects of chronic BPA and ARS exposure on the integrated stress response, we measured HSP70 and HSP27 levels in our chronic/acute stress system. We found no change in HSP70 levels among our treatment conditions (Fig. S3A); a modest but significant increase in HSP27 was observed in our non-chronically treated cells with acute ARS, but was no longer discernible under chronic ARS and chronic BPA treatments (Fig. S3B). Finally, to ensure our chronic treatments were not causing cell death, we examined caspase 3 cleavage (Fig. S4A) and observed general cell growth (Fig. S4B), 20–22 h after recovery from acute stress. We observed no caspase cleavage, indicating apoptotic pathways were not activated. We saw no obvious growth defects attributable to the chronic treatment conditions. The acute BPA treatment does seem to inhibit cell growth, but the effect was observed equally among the chronic treatments.

## DISCUSSION

We show that high doses of BPA trigger the integrated stress response via activation of PERK to induce canonical SGs (Figs 1–3). Besides BPA, we show that high levels of the BPA replacement BPF, the industrial chemical 4-NP, and the pesticide 2,4-D all cause SG assembly (Fig. 4). Together these results indicate that the integrated stress response is triggered by BPA, BPF, 4-NP, and 2,4-D, and indicate a need for more experimentation to define their health effects. Our work adds to a growing body of literature that highlights the physiological effect of BPA, and further indicates a need for caution in its application on everyday products.

Recent public outcry over the adverse health effects of BPA have caused the plastic industry to change its practices, and generate BPA-free products (FDA, 2018). Yet “BPA-free” products are often made using bisphenol derivatives such as BPF and BPS instead of BPA, and there is much less data on their health effects (Eladak et al., 2015). We observed that BPF, but not BPS, causes SG formation (Fig. 4), suggesting that the latter at least for the integrated stress response might be a less problematical substitute for BPA. In any case, caution should be used in replacing BPA with chemicals where less is known regarding their biological effects.

Much BPA research has been centered on its potential endocrine disrupting properties. For example, in the recent CLARITY-BPA study, many of the study endpoints are endocrine-related properties such as ovarian and testicular development, animal growth, and fertility (NTP, 2018). Our results show that while BPA induces SG assembly at high doses, the estrogen mimic β-E does not induce SGs in U2OS cells (Fig. 4). Another industrial compound and endocrine disrupter 4-NP was identified as promoting SG assembly (Fig. 4). U2OS cells do not express estrogen receptors ER-α or ER-β (Tee et al., 2004). While we cannot be certain that ER activation does not trigger SGs in other estrogen-responsive cell types, our data suggest that the mechanisms of BPA and 4-NP SG assembly are independent of their roles in endocrine disruption. Our findings indicate BPA triggers the integrated stress response via PERK activation (Fig. 3; Fig. S1), which aligns with other studies that show an oxidative stress effect of BPA (Gassman, 2017). Oxidative stress and altered SG dynamics have both been implicated in a range of disease states including neurodegenerative diseases (Wolozin, 2012), viral infections (Malinowska et al., 2016), and cancer (Anderson et al., 2015). Whether and how BPA contributes to oxidative stress and cellular damage in these diseases remains to be investigated.

BPA appears to be quickly metabolized with a half-life of about 5 h (Völkel et al., 2008, 2002), and this short half-life could explain why BPA is observed in only 90% of patient samples. A short half-life may also explain the variability we observed in our chronic stress assays, and may require more frequent addition of BPA in our chronic treatment protocols. Interestingly, recent work has found BPA in sweat (Genuis et al., 2012), suggesting that BPA may accumulate in adipose tissue. This could indicate that higher chronic levels could be achieved, and that BPA could also be stored and have high levels in particular tissues. We assessed the effects of chronic physiologically relevant, low nanomolar doses of BPA on cells (Figs 5–7). We observed fewer SGs assembled when long term, low dose BPA treated cells were treated acutely with SG-inducing levels of ARS (Figs 5 and 6). However, stress-induced translational control does not appear to be significantly affected by chronic BPA treatment (Fig. 7), thus uncoupling the physical assembly of SGs from translational control. These data suggest that the preconditioning of cells with low doses of stress actually primes cells to respond differently to acute stress. Altering the ability of a cell to assemble SGs could indicate that the cell is primed to adapt to the stress, which may be a benefit to cell survival. Alternatively, it could indicate that the cell has adapted and no longer considers a stress harmful, which could result in additional cell damage. We did not observe widespread activation of HSP, nor did we observe growth defects or apoptotic cell death attributable to chronic stress treatments (Figs S3 and S4). Still, whether suppression of SG assembly is a benefit or a liability – particularly given that eIF2α-mediated translational control remains intact – remains to be determined. Dampening of the integrated stress response could have a role in neurodegenerative diseases, cancer and viral infection; altering the ability to form SGs may play a role in the influence of environmental factors on the development of these diseases (Costa-Mattioli and Walter, 2020).

Our results suggest that structure alone is not a good indicator of the ability of a compound to induce a stress response (Fig. 4). It is also important to consider that humans currently have contact with as many as 85,000 chemicals obtained from their environments (EPA) some of which may be having toxic effects at their typical exposure levels (Sturla et al., 2014), others of which may be inducing protective effects (Calabrese and Mattson, 2017). It will therefore be critical to continue to examine not only the range of effects of these individual substances, but also the compound effects of substances within the complex chemical context that human cells currently reside. For example, as BPA loses favor with the manufacturing industry due to social and political pressure, the use of BPS, BPF and other chemical analogues will increase. There is already preliminary evidence that bisphenols and other related compounds have synergistic effects that can affect the cellular consequences of exposure (Kortenkamp, 2007; Le Magueresse-Battistoni et al., 2018). The policy implications of this information are profound: it will not be sufficient to continue to make one-off bans on individual chemicals, only to have them replaced by others about which even less is known. The future of ensuring safer environments will depend upon a more holistic understanding of the effects of chemical classes on cellular physiology and human health.

## MATERIALS AND METHODS

### Cell culture and drug treatments

Cells [U2OS (Kedersha et al., 2008), MEF with or without eIF2α-S51A (Scheuner et al., 2001) Hap1 cells (Aulas et al., 2017)] were maintained at 37°C in a CO_2_ incubator in DMEM (Corning) supplemented with 10% FBS, 20 mM Hepes (Gibco), 1% penicillin/streptomycin. Hoechst 33325 staining was periodically performed to check for mycoplasma contamination. For SG induction, cells were treated with chemicals purchased from Sigma-Aldrich including Bisphenol-A (BPA), Bisphenol-S (BPS) Bisphenol-F (BPF), 4-nonylphenol (4-NP), 4-ethylphenol (4-EP), 4-vinylphenol (4-VP), β-estradiol (β-E), sodium arsenite (ARS), and 2,4-dichlorophenoxyacetic acid (2,4-D). As indicated in figure legends, cycloheximide (CHX) and puromycin (puro) were added. For chronic treatments, BPA (5 nM or 10 nM final concentration from 5 µM stock) or mock treatment (methanol) was added when cells were split at the same time every 2–3 days at ∼90% confluency. For 6-day and 1-month BPA or mock treatments, each *n* represents a separate flask of cells split. For Coomassie staining, cells were fixed with 4% paraformaldehyde, then incubated overnight with 0.1% Coomassie Brilliant Blue R250 in water, then rinsed in three washes of water to destain.

### Polysome profiling

5X10^5^ cells were plated onto six-well dishes and the following day treated as indicated. Then cells were treated with 100 µg/ml cycloheximide for 5 min, washed once with HBSS, and 500 µl of polysome buffer (20 mM Tris, pH 7.4, 150 mM NaCl, 5 mM MgCl_2_, 1% Tritonx100, RNaseIN, 1 mM DTT, 100 µg/ml cycloheximide) was added. Cells were scraped and collected into 1.7 ml epi tubes and rotated at 4°C for 10 min and centrifuged at 10,000×***g*** for 10 min at 4°C. The supernatant was loaded onto a preformed 10–50% sucrose gradient made in polysome buffer. Gradients were centrifuged in a Beckman Sw55Ti at 45,000 rpm for 100 min at 4°C. Then a Brandel bottom-piercing apparatus connected to an ISCO UV monitor and pump extracted the gradient while measuring the eluate at 254 nm.

### Immunofluorescence

1×10^5^ U2OS cells were plated into a 24-well dish seed with coverslips. The following day cells were treated as indicated in figure legends. Then the cells were fixed with 4% paraformaldehyde for 15 min, permeabilized with −20°C methanol for 5 min, and blocked for at least 20 min with 5% normal horse serum (NHS; ThermoFisher) diluted in PBS. Primary antibodies were diluted in blocking solution and incubated for 1 h at room temperature or overnight at 4°C. Antibodies against G3BP1(sc-365338), eIF4G (sc-11373), eIF3b (sc-16377), FMRP (sc-101048) FXR1 (sc-10554), TIAR (sc-1749), Hedls (sc-8418) and HuR(sc-5261) were all purchased from Santa Cruz and used at a 1:250 dilution. The DDX6 antibody was purchased from Bethyl Laboratories (A300-461A) and used at a 1:500 dilution. Coverslips were washed three times for 5 min then incubated with secondary antibodies and Hoechst 33258 (Sigma-Aldrich) for 1 h at room temperature and again washed. Coverslips were mounted on glass slides with Vinol.

### Direct fluorescence

7×10^4^ U2OS cells stably expressing GFP-G3BP1, RFP-DCP1 (Kedersha et al., 2008) were plated in 12-well plates seeded with coverslips and 2 days later were treated as indicated. Cells were fixed with 4% paraformaldehyde for 10 min, permeabilized with ice-cold methanol for 10 min, and rinsed with PBS. Hoechst 33258 (Sigma-Aldrich) was used to stain nuclei. Coverslips were mounted on glass slides with Vinol.

### Microscopy

Wide-field fluorescence microscopy was performed using an Eclipse E800 microscope (Nikon) equipped with epifluorescence optics and a digital camera (Spot Pursuit USB) or an AXIO observer A1 (Zeiss) equipped with epifluorescence optics and a digital camera (SPOT Idea 5.0mp). Image acquisition was done with a 40X air or 60X oil objective. Images were merged using Adobe Photoshop.

### siRNA treatment

1.8×10^5^ U2OS cells were seeded in the six-well plates and grown for 2 h, then, transfected using100 pmol siRNA (SmartPool-Dharmacon, Thermo Fisher Scientific), Lipofectamine 2000 (Invitrogen) in OPTI-MEM (Life Technologies). Cells were incubated with siRNA for 24 h at which time a second transfection was done under the same conditions and 24 h later the cells were collected, counted, and plated for experiments.

### Fluorescence *in situ* hybridization (FISH)

U2OS cells were treated as indicated in figure legends then fixed with 4% paraformaldehyde for 15 min, permeabilized with −20°C methanol for 10 min, then dehydrated in 70% ethanol at 4°C. Cells were stored at 4°C for at least overnight then wash several times with 2X saline sodium citrate (SSC; Ambion). Cells were equilibrated in hybridization buffer (Sigma) for 15 min at 65°C then incubated with oligo dT_40X_ probe for 1 h at 42°C. Cells were washed twice with 2X SSC for 10 min at 42°C then several times at room temperature. Coverslips were then processed for immunofluorescences as indicated above (from block step on).

### SG quantification

At least three independent 40X images from at least three independent experiments were counted as the total SG positive over the total number of cells. Cells were counted as SG positive if at least two G3BP1 foci were present in the cytoplasm. The total number of cells was quantified by counting nuclei using Hoechst. Graphs were generated with GraphPad Prism or Excel, and error bars indicate standard deviation or standard error of the mean, as indicated in the figure legend.

### Immunoblotting

5×10^5^ U2OS cells were plated in six-well dishes and the following day drug treated as indicated. Cells were washed once with PBS then lysis buffer [50 mM Hepes pH 7.6, 5% glycerol, 150 mM NaCl, 0.5% NP40, 1X Halt protease inhibitors (Thermo Fisher Scientific), 1X Halt phosphatase inhibitors (Thermo Fisher Scientific)] and cells were scraped and collected into tubes. Cells were sonicated using a cup sonicator and the protein concentration was determined using BioRad Protein Assay (BioRad). SDS dye was added to a 1X concentration and lysates (equal µg of protein) were loaded onto 4–20% Tris-glycine gels (BioRad). Gels were transferred to nitrocellulose, then blocked with 5% milk in TBST. Primary antibodies were diluted in 5% NHS and incubated overnight at 4°C. p-eIF2α (Abcam, ab32157, 1:1000), eIF2α (Cell Signaling Technologies, 2103, 1:1000), GAPDH (Invitrogen, AM4300, 1:5000), HSP70 (StressGen, SPA-810, 1:1000), HSP27 (StressGen, SPA-800, 1:100), Caspase 3 (Cell Signaling Technologies, 9662-S, 1:1000) or puromycin (EMD Millipore, MABE343, 1:500). Secondary antibodies were diluted 1:10,000 in 5% NHS and incubated for 1 h at room temperature. Blots were washed at least three times for 10 min after antibody incubation. HRP signal detected using SuperSignal West Pico Chemiluminescent Substrate (Thermo Fisher Scientific),

### Molecular structures

Molecular structures were generated with ChemSketch by ACD Labs Freeware 2016.

### Statistical analysis

Statistical analysis of an unpaired *t*-test was performed where indicated and statistical significance was considered *P*<0.05. *n* indicates the number of replicate experiments and within each experiment and at least 100 cells were counted. Statistical analysis of western blot quantifications were performed by two-way or one-way ANOVA, as indicated, followed by pairwise comparisons of sample means by the Tukey HSD test, using VassarStats open source analytical tools (vassarstats.net).

## Supplementary Material

Supplementary information

## References

[BIO057539C1] Anderson P. and Kedersha N. (2009). Stress granules. *Curr. Biol.* 19, R397-R398. 10.1016/j.cub.2009.03.013 19467203

[BIO057539C2] Anderson P., Kedersha N. and Ivanov P. (2015). Stress granules, P-bodies and cancer. *Biochim. Biophys. Acta* 1849, 861-870. 10.1016/j.bbagrm.2014.11.009 25482014 PMC4457708

[BIO057539C3] Apau J., Acheampong A. and Adua E (2018). Exposure to Bisphenol A, Bisphenol F, and Bisphenol S can result in obesity in human body. *Cogent Chem.* 4, 1-7. 10.1080/23312009.2018.1506601

[BIO057539C4] Arimoto K., Fukuda H., Imajoh-Ohmi S., Saito H. and Takekawa M. (2008). Formation of stress granules inhibits apoptosis by suppressing stress-responsive MAPK pathways. *Nat. Cell Biol.* 10, 1324-1332. 10.1038/ncb1791 18836437

[BIO057539C5] Asimakopoulos A. G. and Thomaidis N. S. (2015). Bisphenol A, 4-t-octylphenol, and 4-nonylphenol determination in serum by hybrid solid phase extraction-precipitation technology technique tailored to liquid chromatography-tandem mass spectrometry. *J. Chromatogr. B Analyt. Technol. Biomed. Life Sci.* 986-987, 85-93. 10.1016/j.jchromb.2015.02.009 25725318

[BIO057539C6] Aulas A., Fay M. M., Lyons S. M., Achorn C. A., Kedersha N., Anderson P. and Ivanov P. (2017). Stress-specific differences in assembly and composition of stress granules and related foci. *J. Cell Sci.* 130, 927-937. 10.1242/jcs.199240 28096475 PMC5358336

[BIO057539C7] Ben-Jonathan N. (2019). Endocrine disrupting chemicals and breast cancer: the saga of bisphenol a. In *Estrogen Receptor and Breast Cancer: Celebrating the 60th Anniversary of the Discovery of ER* (ed. Zhang X.), pp. 343-377 Cham: Springer International Publishing.

[BIO057539C8] Buchan J. R., Muhlrad D. and Parker R. (2008). P bodies promote stress granule assembly in Saccharomyces cerevisiae. *J. Cell Biol.* 183, 441-455. 10.1083/jcb.200807043 18981231 PMC2575786

[BIO057539C9] Calabrese E. J. and Mattson M. P. (2017). How does hormesis impact biology. toxicology, and medicine? *NPJ Aging Mech Dis* 3, 13 10.1038/s41514-017-0013-z 28944077 PMC5601424

[BIO057539C10] Calafat A. M., Kuklenyik Z., Reidy J. A., Caudill S. P., Ekong J. and Needham L. L. (2005). Urinary concentrations of Bisphenol A and 4-nonylphenol in a human reference population. *Environ. Health Perspect.* 113, 391-395. 10.1289/ehp.7534 15811827 PMC1278476

[BIO057539C11] Calafat A. M., Ye X., Wong L. Y., Reidy J. A. and Needham L. L. (2008). Exposure of the U.S. population to Bisphenol A and 4-tertiary-octylphenol: 2003-2004. *Environ. Health Perspect.* 116, 39-44. 10.1289/ehp.10753 18197297 PMC2199288

[BIO057539C12] Costa-Mattioli M. and Walter P. (2020). The integrated stress response: from mechanism to disease. *Science* 368, eaat5314 10.1126/science.aat5314 32327570 PMC8997189

[BIO057539C13] Eladak S., Grisin T., Moison D., Guerquin M. J., N'tumba-Byn T., Pozzi-Gaudin S., Benachi A., Livera G., Rouiller-Fabre V. and Habert R. (2015). A new chapter in the Bisphenol A story: Bisphenol S and Bisphenol F are not safe alternatives to this compound. *Fertil. Steril.* 103, 11-21. 10.1016/j.fertnstert.2014.11.005 25475787

[BIO057539C14] Epa *TSCA Chemical Substance Inventory* [Online] Available: https://www.epa.gov/tsca-inventory/about-tsca-chemical-substance-inventory [Accessed February 5, 2019 2019].

[BIO057539C15] Farny N. G., Kedersha N. L. and Silver P. A. (2009). Metazoan stress granule assembly is mediated by P-eIF2alpha-dependent and -independent mechanisms. *RNA* 15, 1814-1821. 10.1261/rna.1684009 19661161 PMC2743051

[BIO057539C16] FDA (2018). Bisphenol A (BPA): Use in Food Contact Application. In: FDA (ed.).

[BIO057539C17] Garcia M. A., Gil J., Ventoso I., Guerra S., Domingo E., Rivas C. and Esteban M. (2006). Impact of protein kinase PKR in cell biology: from antiviral to antiproliferative action. *Microbiol. Mol. Biol. Rev.* 70, 1032-1060. 10.1128/MMBR.00027-06 17158706 PMC1698511

[BIO057539C18] Gassman N. R. (2017). Induction of oxidative stress by Bisphenol A and its pleiotropic effects. *Environ. Mol. Mutagen.* 58, 60-71. 10.1002/em.22072 28181297 PMC5458620

[BIO057539C19] Genuis S. J., Beesoon S., Birkholz D. and Lobo R. A. (2012). Human excretion of Bisphenol A: blood, urine, and sweat (BUS) study. *J. Environ. Public Health* 2012, 185731 10.1155/2012/185731 22253637 PMC3255175

[BIO057539C20] Kedersha N. L., Gupta M., Li W., Miller I. and Anderson P. (1999). RNA-binding proteins TIA-1 and TIAR link the phosphorylation of eIF-2α to the assembly of mammalian stress granules. *J. Cell Biol.* 147, 1431-1442. 10.1083/jcb.147.7.1431 10613902 PMC2174242

[BIO057539C21] Kedersha N., Cho M. R., Li W., Yacono P. W., Chen S., Gilks N., Golan D. E. and Anderson P. (2000). Dynamic shuttling of TIA-1 accompanies the recruitment of mRNA to mammalian stress granules. *J. Cell Biol.* 151, 1257-1268. 10.1083/jcb.151.6.1257 11121440 PMC2190599

[BIO057539C22] Kedersha N., Chen S., Gilks N., Li W., Miller I. J., Stahl J. and Anderson P. (2002). Evidence that ternary complex (eIF2-GTP-tRNA(i)(Met))-deficient preinitiation complexes are core constituents of mammalian stress granules. *Mol. Biol. Cell* 13, 195-210. 10.1091/mbc.01-05-0221 11809833 PMC65082

[BIO057539C23] Kedersha N., Stoecklin G., Ayodele M., Yacono P., Lykke-Andersen J., Fritzler M. J., Scheuner D., Kaufman R. J., Golan D. E. and Anderson P. (2005). Stress granules and processing bodies are dynamically linked sites of mRNP remodeling. *J. Cell Biol.* 169, 871-884. 10.1083/jcb.200502088 15967811 PMC2171635

[BIO057539C24] Kedersha N., Tisdale S., Hickman T. and Anderson P (2008). Real-time and quantitative imaging of mammalian stress granules and processing bodies. *Methods Enzymol.* 448, 521-552. 10.1016/S0076-6879(08)02626-8 19111193

[BIO057539C25] Kedersha N., Panas M. D., Achorn C. A., Lyons S., Tisdale S., Hickman T., Thomas M., Lieberman J., Mcinerney G. M., Ivanov P. et al. (2016). G3BP-Caprin1-USP10 complexes mediate stress granule condensation and associate with 40S subunits. *J. Cell Biol.* 212, 845-860. 10.1083/jcb.201508028 27022092 PMC4810302

[BIO057539C26] Kortenkamp A. (2007). Ten years of mixing cocktails: a review of combination effects of endocrine-disrupting chemicals. *Environ. Health Perspect.* 115, 98-105. 10.1289/ehp.9357 18174957 PMC2174407

[BIO057539C27] Langos D. and Granvogl M. (2016). Studies on the simultaneous formation of aroma-active and toxicologically relevant vinyl aromatics from free phenolic acids during wheat beer brewing. *J. Agric. Food Chem.* 64, 2325-2332. 10.1021/acs.jafc.5b05606 26800353

[BIO057539C28] Le Magueresse-Battistoni B., Vidal H. and Naville D (2018). Environmental pollutants and metabolic disorders: the multi-exposure scenario of life. *Front. Endocrinol.* 9, 582 10.3389/fendo.2018.00582 PMC6176085 30333793

[BIO057539C29] Li X., Ying G.-G., Zhao J.-L., Chen Z.-F., Lai H.-J. and Su H.-C. (2013). 4-Nonylphenol, Bisphenol-A and triclosan levels in human urine of children and students in China, and the effects of drinking these bottled materials on the levels. *Environ. Int.* 52, 81-86. 10.1016/j.envint.2011.03.026 21794921

[BIO057539C30] Malinowska M., Niedzwiedzka-Rystwej P., Tokarz-Deptula B. and Deptula W. (2016). Stress granules (SG) and processing bodies (PB) in viral infections. *Acta Biochim. Pol.* 63, 183-188. 10.18388/abp.2015_1060 26894234

[BIO057539C31] McEwen E., Kedersha N., Song B., Scheuner D., Gilks N., Han A., Chen J.-J., Anderson P. and Kaufman R. J. (2005). Heme-regulated inhibitor kinase-mediated phosphorylation of eukaryotic translation initiation factor 2 inhibits translation, induces stress granule formation, and mediates survival upon arsenite exposure. *J. Biol. Chem.* 280, 16925-16933. 10.1074/jbc.M412882200 15684421

[BIO057539C32] Ntp, (2018). NTP Research Report on the CLARITY-BPA Core Study: A Perinatal and Chronic Extended-Dose-Range Study of Bisphenol A in Rats. In: PROGRAM, N. T. (ed.) Research Triangle Park, NC. 31305969

[BIO057539C33] Pakos-Zebrucka K., Koryga I., Mnich K., Ljujic M., Samali A. and Gorman A. M. (2016). The integrated stress response. *EMBO Rep.* 17, 1374-1395. 10.15252/embr.201642195 27629041 PMC5048378

[BIO057539C34] Pfeifer D., Chung Y. M. and Hu M. C. (2015). Effects of low-dose Bisphenol A on DNA damage and proliferation of breast cells: the role of c-Myc. *Environ. Health Perspect.* 123, 1271-1279. 10.1289/ehp.1409199 25933419 PMC4671234

[BIO057539C35] Provvisiero D. P., Pivonello C., Muscogiuri G., Negri M., De Angelis C., Simeoli C., Pivonello R. and Colao A. (2016). Influence of Bisphenol A on type 2 diabetes mellitus. *Int. J. Environ. Res. Public Health* 13, 989 10.3390/ijerph13100989 PMC5086728 27782064

[BIO057539C36] Qiu W., Zhao Y., Yang M., Farajzadeh M., Pan C. and Wayne N. L. (2016). Actions of Bisphenol A and Bisphenol S on the reproductive neuroendocrine system during early development in zebrafish. *Endocrinology* 157, 636-647. 10.1210/en.2015-1785 26653335

[BIO057539C37] Rochester J. R. (2013). Bisphenol A and human health: a review of the literature. *Reprod. Toxicol.* 42, 132-155. 10.1016/j.reprotox.2013.08.008 23994667

[BIO057539C38] Rosenmai A. K., Dybdahl M., Pedersen M., Alice Van Vugt-Lussenburg B. M., Wedebye E. B., Taxvig C. and Vinggaard A. M. (2014). Are structural analogues to bisphenol a safe alternatives? *Toxicol. Sci.* 139, 35-47. 10.1093/toxsci/kfu030 24563381

[BIO057539C39] Scheuner D., Song B., Mcewen E., Liu C., Laybutt R., Gillespie P., Saunders T., Bonner-Weir S. and Kaufman R. J. (2001). Translational control is required for the unfolded protein response and in vivo glucose homeostasis. *Mol. Cell.* 7, 1165-1176. 10.1016/s1097-2765(01)00265-9. PMID: 11430820 11430820

[BIO057539C40] Seachrist D. D., Bonk K. W., Ho S. M., Prins G. S., Soto A. M. and Keri R. A. (2016). A review of the carcinogenic potential of bisphenol A. *Reprod. Toxicol.* 59, 167-182. 10.1016/j.reprotox.2015.09.006 26493093 PMC4783235

[BIO057539C41] Sonenberg N. and Hinnebusch A. G. (2009). Regulation of translation initiation in eukaryotes: mechanisms and biological targets. *Cell* 136, 731-745. 10.1016/j.cell.2009.01.042 19239892 PMC3610329

[BIO057539C42] Sturla S. J., Boobis A. R., Fitzgerald R. E., Hoeng J., Kavlock R. J., Schirmer K., Whelan M., Wilks M. F. and Peitsch M. C. (2014). Systems toxicology: from basic research to risk assessment. *Chem. Res. Toxicol.* 27, 314-329. 10.1021/tx400410s 24446777 PMC3964730

[BIO057539C43] Tee M. K., Rogatsky I., Tzagarakis-Foster C., Cvoro A., An J., Christy R. J., Yamamoto K. R. and Leitman D. C. (2004). Estradiol and selective estrogen receptor modulators differentially regulate target genes with estrogen receptors α and β. *Mol. Biol. Cell* 15, 1262-1272. 10.1091/mbc.e03-06-0360 14699072 PMC363122

[BIO057539C44] Teixeira R., Dopico-García S., Andrade P. B., Valentão P., López-Vilariño J. M., González-Rodríguez V., Cela-Pérez C. and Silva L. R. (2015). Volatile phenols depletion in red wine using molecular imprinted polymers. *J. Food Sci. Technol.* 52, 7735-7746. 10.1007/s13197-015-1892-2 26604347 PMC4648907

[BIO057539C45] Völkel W., Colnot T., Csanady G. A., Filser J. G. and Dekant W. (2002). Metabolism and kinetics of bisphenol a in humans at low doses following oral administration. *Chem. Res. Toxicol.* 15, 1281-1287. 10.1021/tx025548t 12387626

[BIO057539C46] Völkel W., Kiranoglu M. and Fromme H. (2008). Determination of free and total bisphenol A in human urine to assess daily uptake as a basis for a valid risk assessment. *Toxicol. Lett.* 179, 155-162. 10.1016/j.toxlet.2008.05.002 18579321

[BIO057539C47] Wolozin B. (2012). Regulated protein aggregation: stress granules and neurodegeneration. *Mol. Neurodegener* 7, 56 10.1186/1750-1326-7-56 23164372 PMC3519755

