# Supplementary material for: Bisphenol A promotes stress granule assembly and modulates the integrated stress response
Source: Biol Open. 2021 Jan 11;10(1):bio057539. doi: 10.1242/bio.057539 (PMC7823164; doi:10.1242/bio.057539)
Supplement: Supplementary information [file biolopen-10-057539-s1.pdf]

Figure S1

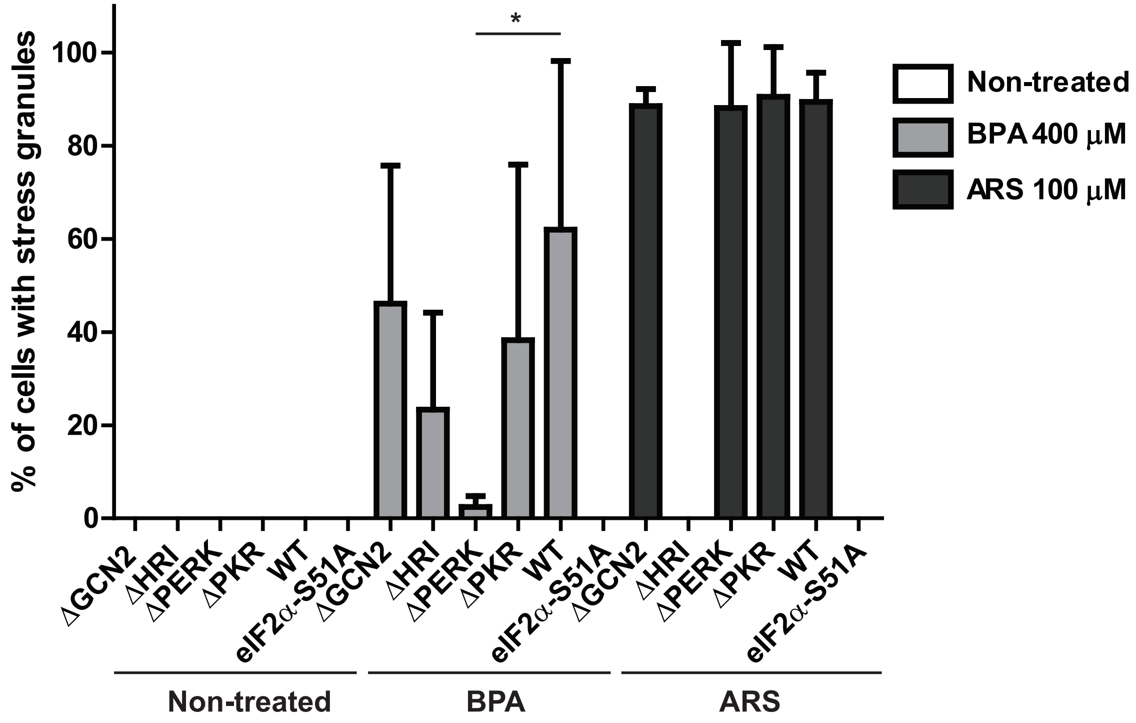

Figure S1: PERK activation is required for the SG response to BPA

Quantification of SGs detected by immunofluorescence in Hap1 cells that were genetically modified with CRISPR to knockout GCN2, HRI, PERK, PKR, or to contain the point mutation eIF2α-S51A. Cells were treated with BPA or ARS for 1 hour, or left untreated. Graph represent means with standard deviation, n=3, * indicates $p<0.05$.

Biology Open • Supplementary information

Figure S2

A

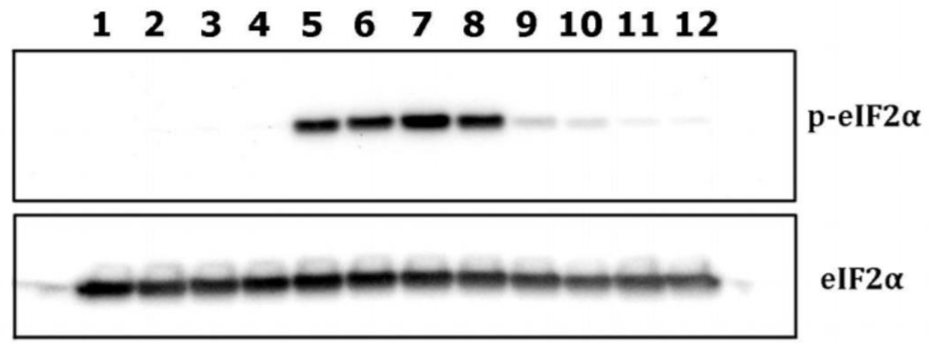

B

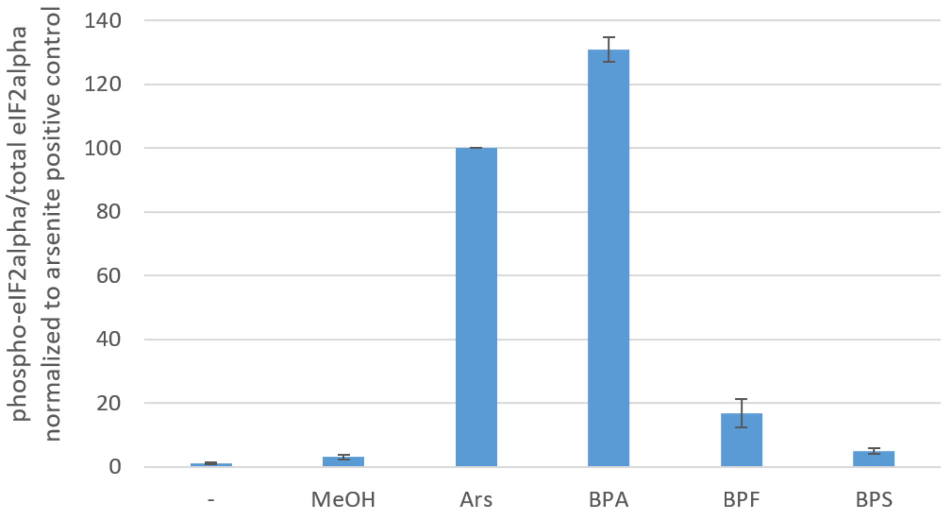

Figure S2: Western blot analysis of bisphenol treated cells.

A) U2OS extracts blotted for phosphorylated eIF2α (top) and total eIF2α (bottom). Lanes: untreated (1 and 2), methanol vehicle (3 and 4), 500 μM arsenite (5 and 6), 500 μM BPA (7 and 8), 500 μM BPF (9 and 10), and 500 μM BPS (11 and 12).

B) Quantification of western blot results. Error bars are +/- S.E.M., n=3.

Biology Open • Supplementary information

Figure S3

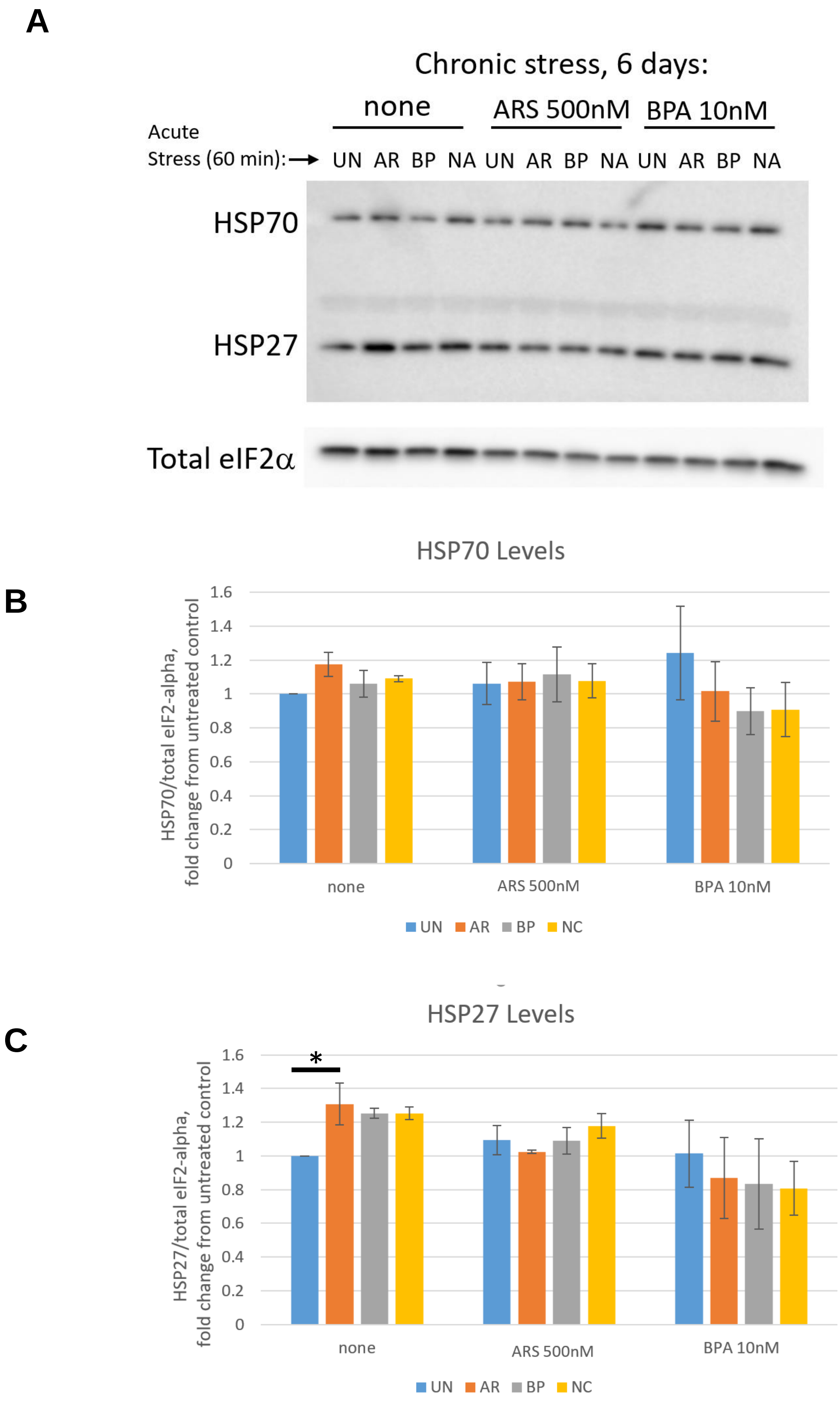

Biology Open • Supplementary information

Figure S3: Heat shock protein levels in response to acute and chronic stress. A) Western blotting of U2OS cells treated with chronic stress for 6 days (none, 500nM arsenite, 10nM BPA), followed by acute stress treatments with 100 μM arsenite (AR, orange bars), 400 μM BPA (BP, grey bars) or 0.2M sodium chloride (NA, yellow bars), or untreated (UN, blue bars), with antibodies to HSP70, HSP27, and total eIF2α. B-C) Quantification of the HSP70 (B) and HSP27 (C) blots relative to total eIF2α. n=3, error bars are +/- S.E.M. * $p<0.05$ by one-way ANOVA within chronic treatment groups and Tukey HSD test.

Biology Open • Supplementary information

Figure S4

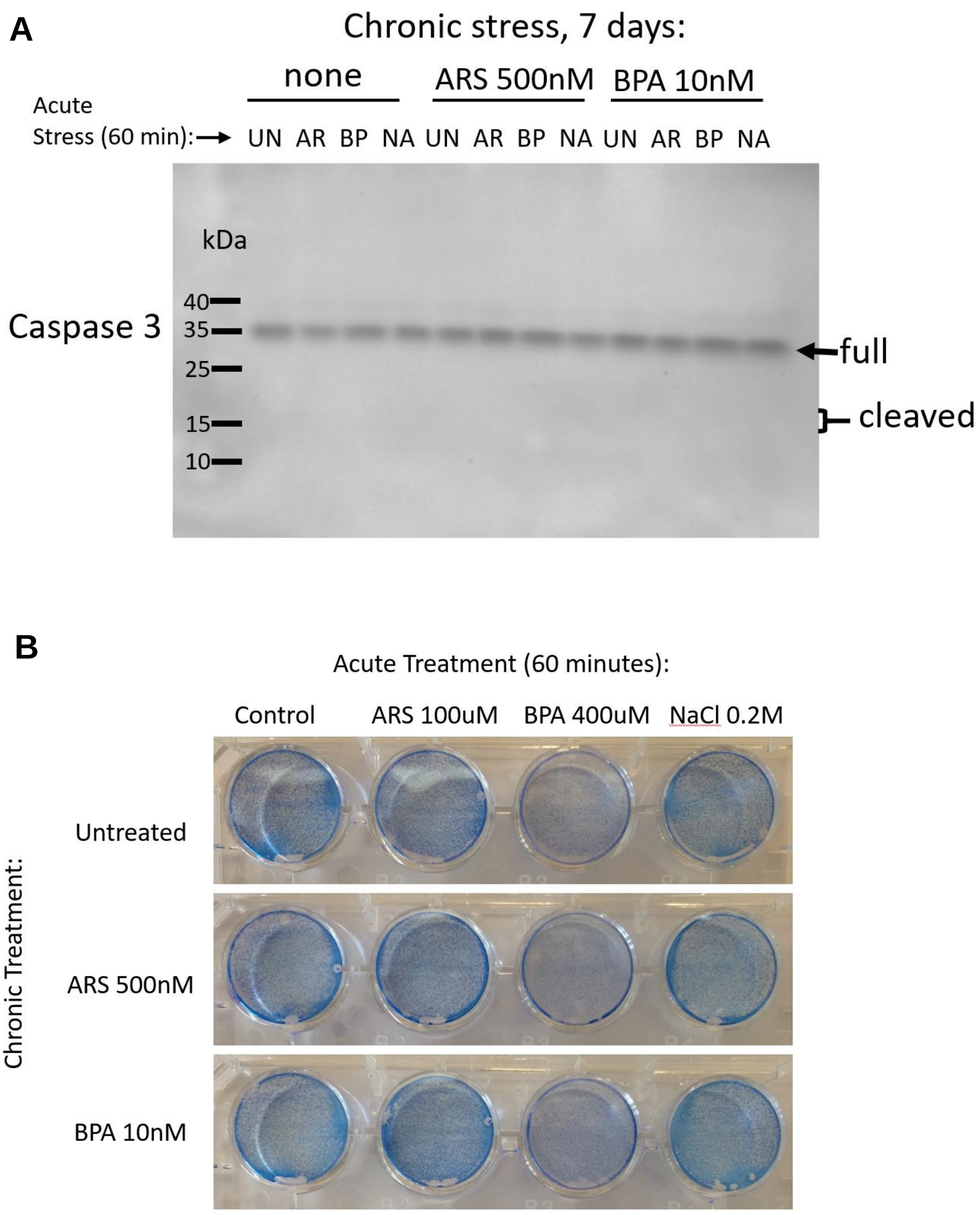

Biology Open • Supplementary information

Figure S4: Chronic stress does not cause apoptotic cell death or significant growth suppression. A) U2OS cells treated as in Figure 7 and Figure S3 were permitted to recover under chronic stress conditions for 20-22 hours after the acute treatment on day 6, and then were harvested on day 7 for western blotting using an antibody that detects both full-length and cleaved Caspase 3. B) Cells treated as described in A were fixed and stained with Coomassie to visualize cell density. Images shown are representative of three experimental replicates.
